# Ductus Arteriosus Morphology in Pulmonary Atresia With Ventricular Septal Defect: A Retrospective Case Series Comparing Echocardiography and Digital Subtraction Angiography

**DOI:** 10.7759/cureus.108765

**Published:** 2026-05-13

**Authors:** Tien T Quan, Hai M Nguyen, Hao T Nguyen, Bao An H Nguyen

**Affiliations:** 1 University of Health Sciences, Vietnam National University Ho Chi Minh City, Ho Chi Minh, VNM; 2 Department of Cardiology, Children’s Hospital 1, Ho Chi Minh, VNM

**Keywords:** congenital heart disease, digital subtraction angiography, ductus arteriosus, echocardiography, pulmonary atresia ventricular septal defect

## Abstract

Objective: This study aims to describe ductus arteriosus (DA) morphology in patients with pulmonary atresia with ventricular septal defect (PA-VSD) and to assess exploratory paired differences between echocardiography and digital subtraction angiography (DSA). This study was primarily descriptive and was not designed to establish diagnostic agreement, equivalence, or interchangeability between the two imaging modalities. Materials and methods: This retrospective descriptive case series was conducted at Children’s Hospital 1, Ho Chi Minh City, Vietnam, from April 2024 to April 2025. Consecutive patients aged from birth to under 18 years with confirmed PA-VSD, a patent DA, and available echocardiography and DSA images adequately showing DA morphology were included. PA-VSD was confirmed by echocardiographic evidence of pulmonary atresia, a ventricular septal defect, and absence of antegrade flow from the right ventricle to the pulmonary arteries, with further verification by DSA and catheterization records. Patients with incomplete records, inadequate images, or cardiovascular anomalies substantially altering ductal anatomy were excluded. DA origin, orientation, number, and diameters at the aortic and pulmonary ends were retrospectively remeasured from archived images and independently assessed for each modality. Paired statistical tests were used to evaluate modality-related differences, and exact concordance was described for categorical findings.

Results: Thirty patients were included. The median age at admission was 92 days, and the median body weight was 3.6 kg. There were 20 female patients (66.7%) and 10 male patients (33.3%). PGE1 infusion was administered in 22 patients (73.3%). All patients had a single DA on both modalities. On echocardiography, DA origin was classified as the descending aorta in 16 patients (53.3%), the brachiocephalic artery in 11 patients (36.7%), and the subclavian artery in three patients (10.0%). On DSA, the DA origin was classified as the descending aorta in 15 patients (50.0%), the brachiocephalic artery in 12 patients (40.0%), and the subclavian artery in three patients (10.0%). The distribution of DA origin did not differ significantly between modalities (McNemar-Bowker test, χ² = 1.00, p = 0.608), but this result was not interpreted as evidence of agreement. Vertical orientation was observed in 17 patients (56.7%) on both modalities, with exact concordance for DA orientation and DA number. The mean aortic-end diameter was significantly larger on DSA than on echocardiography (3.86 ± 1.43 mm vs. 3.12 ± 0.97 mm; paired-samples t-test, t = 3.18, p = 0.001). The mean pulmonary-end diameter did not differ significantly between DSA and echocardiography (1.98 ± 0.83 mm vs. 2.18 ± 0.62 mm; t = 1.32, p = 0.198).

Conclusions: DA morphology in PA-VSD was variable, with descending aortic origin and vertical orientation being common. Echocardiography and DSA showed similar categorical distributions for DA origin, orientation, and number, but these findings do not establish diagnostic agreement or interchangeability. DSA demonstrated larger aortic-end measurements, although the clinical impact of this difference on stent sizing, procedural success, or complications was not directly evaluated. Future studies should assess agreement, reproducibility, and procedural outcomes using standardized imaging protocols.

## Introduction

Pulmonary atresia with ventricular septal defect (PA-VSD) is a rare and complex cyanotic congenital heart disease in which pulmonary blood flow is highly variable. It may depend on the ductus arteriosus (DA), major aortopulmonary collateral arteries (MAPCAs), or both. Anatomical and functional differences of the DA have been described in patients with pulmonary atresia with and without a ventricular septal defect, highlighting its clinical relevance in this condition [1].

The DA plays an important role in maintaining pulmonary perfusion in duct-dependent congenital heart disease. Pharmacological maintenance of ductal patency using prostaglandin E1 may be used as an initial stabilizing measure in neonates and infants with duct-dependent cardiac lesions [2]. After stabilization, treatment options may include ductal stenting, creation of a systemic-to-pulmonary shunt, or other staged palliative strategies, depending on patient status and anatomical suitability [3,4].

DA morphology in PA-VSD may differ from that in other duct-dependent lesions because the pulmonary arteries are not supplied through a normal right ventricular outflow tract. As a result, the DA may arise from different sites of the aortic arch or its branches, follow a vertical or tortuous course, and insert into the central or branch pulmonary arteries. These anatomical variations are clinically important because they may influence the stability of pulmonary blood flow and the feasibility of catheter-based or surgical palliation [1,5].

The morphology of the DA is particularly relevant in patients being considered for ductal stenting. DA origin may influence the choice of vascular access and catheter trajectory, whereas ductal course, angulation, tortuosity, and diameter may affect guidewire passage, stent positioning, ductal coverage, and procedural stability. A sharply angulated, elongated, tortuous, or unusually originated DA may increase procedural difficulty and may contribute to complications such as incomplete ductal coverage, stent migration, vascular injury, or residual obstruction. Comparative studies have also evaluated ductal stenting and modified Blalock-Taussig shunts in relation to pulmonary artery growth and subsequent surgical planning [6].

Echocardiography is commonly used as the initial imaging modality in PA-VSD because it is non-invasive, widely available, and provides bedside information on intracardiac anatomy, ventricular function, ductal flow, and pulmonary artery anatomy. However, complete delineation of extracardiac vascular structures may be limited by acoustic windows, patient size, ductal orientation, and complex arch or branch pulmonary artery anatomy. CT angiography and advanced echocardiographic techniques have increasingly improved non-invasive anatomical assessment. Still, digital subtraction angiography (DSA) remains clinically relevant in selected patients because it provides real-time, high-resolution angiographic visualization during catheterization and can directly guide interventional planning. DSA may be particularly useful when a precise definition of DA origin, course, pulmonary insertion, and vessel caliber is required before ductal stenting or surgical decision-making [7].

Previous studies have described anatomical and functional differences of the DA in pulmonary atresia with and without ventricular septal defect, angiographic classifications of ductal morphology, and the influence of ductal anatomy on interventional outcomes [1,5,7]. However, direct paired assessments of DA morphology using echocardiography and DSA remain limited, particularly in regional pediatric cohorts. In Vietnam, data specifically describing DA morphology in PA-VSD are scarce, and few studies have provided quantitative measurements of DA origin, orientation, number, and diameter using paired imaging modalities. This creates a practical knowledge gap for clinicians deciding whether echocardiography provides sufficient initial anatomical information or whether invasive angiographic assessment is required for procedural planning.

Therefore, this study aimed to describe the morphological characteristics of the DA in patients with PA-VSD and to assess, in an exploratory manner, paired differences between echocardiography and DSA. The study was primarily descriptive and was not designed to establish diagnostic equivalence, formal agreement, or interchangeability between the two imaging modalities.

## Materials and methods

Study design and setting

This retrospective descriptive case series was conducted at the Department of Cardiology, Children’s Hospital 1, Ho Chi Minh City, Vietnam, from April 2024 to April 2025. The study was primarily descriptive, with an exploratory paired assessment comparing echocardiography and DSA. It was not designed to establish diagnostic equivalence, formal agreement, or interchangeability between the two imaging modalities.

Study population

The study population included pediatric patients aged from birth to under 18 years who were diagnosed with PA-VSD and admitted to Children’s Hospital 1 during the study period. PA-VSD was confirmed based on echocardiographic evidence of pulmonary valve or right ventricular outflow tract atresia, the presence of a ventricular septal defect, and the absence of antegrade flow from the right ventricle to the pulmonary arteries. DSA findings and catheterization records further supported diagnostic confirmation. The pediatric cardiology team reviewed all cases to confirm eligibility.

Inclusion and exclusion criteria

Patients were included if they met all of the following criteria: age from birth to under 18 years, confirmed diagnosis of PA-VSD, presence of a patent DA, and availability of both echocardiography and DSA images with adequate visualization of DA morphology.

Patients were excluded if they were 18 years of age or older, had incomplete medical records with more than 20% missing data, had inadequate echocardiographic or angiographic images preventing reliable assessment of DA morphology, or had associated major cardiovascular anomalies that could substantially alter ductal or aortic arch anatomy, including aortic coarctation, aortic arch hypoplasia, interrupted aortic arch, or aortic atresia.

One patient with associated transposition of the great arteries was retained in the analysis because this anomaly did not prevent identification of DA origin, course, number, or diameters on either echocardiography or DSA and was not considered to substantially alter DA morphology for this study.

All consecutive eligible patients who underwent both echocardiography and DSA during the study period and met the inclusion criteria were included to reduce selection bias. Because this was a retrospective study based on available paired echocardiographic and DSA data, the total number of PA-VSD patients initially screened during the study period, and the number excluded before the availability of paired imaging, could not be reliably reconstructed from the retrospective dataset.

MAPCAs and associated anatomical assessment

No patient with confirmed MAPCAs was included in the final analysis. MAPCAs were reviewed on available DSA images and catheterization records and were differentiated from the DA based on origin, course, pulmonary insertion, and angiographic appearance.

Additional anatomical characteristics relevant to PA-VSD severity, including pulmonary artery confluence, right-sided aortic arch, and branch pulmonary artery hypoplasia, were reviewed when available. However, these variables were not consistently documented in the retrospective dataset and were therefore not included as primary analytical variables.

Data collection and measurements

Data were retrospectively extracted from medical records using standardized data collection forms. Collected variables included demographic characteristics, clinical presentation, prostaglandin E1 (PGE1) exposure, peripheral oxygen saturation (SpO₂) before and after PGE1 administration, timing of imaging studies when available, and detailed morphological features of the DA.

Morphological characteristics included DA origin, orientation, number, and diameters at the aortic and pulmonary ends. The DA origin was categorized as arising from the brachiocephalic artery, subclavian artery, or descending aorta. Measurements were obtained by retrospective review of archived echocardiographic and DSA images specifically for this study, rather than being extracted solely from the original written imaging reports.

DA diameters were measured at the aortic and pulmonary ends using an inner-edge-to-inner-edge method. Echocardiographic measurements were taken from the imaging plane that best visualized the ductal lumen and its connection to the systemic and pulmonary arterial circulation. DSA measurements were obtained from calibrated angiographic images during optimal ductal opacification. When multiple frames were available, the frame with the clearest luminal border and least vessel overlap was selected. Because of the retrospective design, the cardiac phase could not be standardized across all archived images.

DA measurements were reported as absolute diameters in millimeters and were not indexed to body weight or body surface area. This approach was used because the study aimed to describe ductal dimensions relevant to anatomical assessment and procedural planning; however, the absence of indexed measurements was accounted for when interpreting the results.

Definition of DA orientation

DA orientation was classified as vertical when the main ductal axis formed an angle ≥45° relative to the horizontal plane of the aortic arch and horizontal when the angle was <45°. This cutoff was a pragmatic operational definition developed for this study to standardize retrospective classification across modalities. Because the ≥45° cutoff has not been externally validated, orientation results were interpreted descriptively rather than as a validated anatomical classification.

DA orientation was independently classified as vertical or horizontal on echocardiography and DSA using the same predefined cutoffs. DSA was not used as a reference modality for assigning echocardiographic orientation.

Imaging assessment

All patients underwent both echocardiography and DSA. Echocardiography was performed as the initial non-invasive imaging modality, while DSA was used to further delineate DA anatomy and support procedural planning.

Echocardiographic assessment focused on intracardiac anatomy, confirmation of PA-VSD, ductal patency, DA origin, DA course, pulmonary arterial insertion, DA number, and DA diameters at the aortic and pulmonary ends.

DSA was performed in the catheterization laboratory by the pediatric interventional cardiology team. Angiographic assessment was obtained after contrast injection into the aortic arch and/or relevant systemic arterial branches. Standard angiographic projections included anteroposterior and lateral views, with additional oblique projections obtained as needed to delineate the DA origin, course, pulmonary insertion, pulmonary artery confluence, and branch pulmonary artery anatomy. Catheter position, injection site, and projection angle were selected based on the suspected ductal origin and patient anatomy.

Sedation or anesthesia during DSA was administered according to institutional catheterization laboratory protocols. Because sedation, anesthesia, and hemodynamic conditions may influence vascular tone and vessel caliber, this factor was considered when interpreting DSA-based ductal diameter measurements.

Imaging timing and PGE1 exposure

PGE1 administration status was extracted from medical records. The timing of echocardiography and DSA relative to PGE1 infusion was recorded when available because PGE1 may affect DA patency, ductal caliber, and morphology. The interval between echocardiography and DSA was also recorded when available to assess the possibility of temporal changes in DA morphology between modalities. Post-PGE1 SpO₂ values were summarized only among patients who received PGE1 infusion.

Image reviewers and discrepancy resolution

Echocardiographic images were reviewed by a pediatric cardiologist with 12 years of experience in echocardiography of congenital heart disease. DSA images were reviewed by one pediatric interventional cardiologist with 13 years of experience in congenital cardiac catheterization.

Image review was performed separately for each modality. Complete blinding to findings from the alternate modality could not be guaranteed because of the retrospective design and because both imaging studies were used clinically during patient care. Discrepancies between echocardiography and DSA were not forced into a single final classification for comparative analyses; instead, modality-specific findings were retained and reported descriptively. Because only one reviewer interpreted each modality, interobserver agreement could not be assessed.

Sample size

Because this was a retrospective descriptive case series of a rare congenital heart disease, no formal sample size calculation was performed. All consecutive eligible patients with confirmed PA-VSD who underwent both echocardiography and DSA during the study period and met the inclusion criteria were included. The final sample consisted of 30 patients.

Statistical analysis

Data entry was performed using EpiData software (EpiData Association, Odense, Denmark), and statistical analyses were conducted using Stata version 17 (StataCorp LLC, College Station, TX, US). Continuous variables were assessed for distributional characteristics using visual inspection and the Shapiro-Wilk test. Normally distributed continuous variables were presented as mean ± standard deviation, while skewed variables were presented as median and interquartile range. Categorical variables were summarized as frequencies and percentages.

Because echocardiography and DSA findings were obtained from the same patients, paired statistical tests were used. For DA diameter measurements, the assumption for the paired-samples t-test was assessed using the distribution of paired differences between DSA and echocardiography rather than the distribution of raw measurements alone. Paired continuous variables were compared using the paired-samples t-test when paired differences were approximately normally distributed; otherwise, the Wilcoxon signed-rank test was used.

For paired categorical variables, McNemar’s test was used for binary variables, and the Stuart-Maxwell or McNemar-Bowker test was used for variables with more than two categories. Variables with no variation across modalities, such as DA number when all patients had a single DA on both modalities, were described without formal hypothesis testing.

In addition to hypothesis testing, exact concordance and discordant case counts were reported for paired categorical findings when applicable. Formal diagnostic agreement analyses, including Cohen’s kappa, Bland-Altman analysis, and intraclass correlation coefficients, were not performed as primary analyses. Therefore, non-significant p-values were not interpreted as evidence of diagnostic agreement, equivalence, or interchangeability between modalities.

All tests were two-sided, and p-values <0.05 were considered statistically significant.

Ethical considerations

The study was approved by the University of Health Sciences, Vietnam National University, Ho Chi Minh City, under approval number C2023-44-04. Because this was a retrospective review of de-identified clinical and imaging data, the requirement for informed consent for study participation was waived. All procedures were conducted in accordance with institutional ethical standards and applicable regulations. The data were stored as de-identified participant data and were available from the corresponding author upon reasonable request.

## Results

From April 2024 to April 2025, 30 consecutive eligible patients with confirmed PA-VSD were included in the final analysis. All included patients had a patent DA that could be evaluated on both echocardiography and DSA.

Demographic and clinical characteristics

Demographic and clinical characteristics of the 30 included patients are presented in Table 1.

**Table 1:** Demographic and clinical characteristics of the study population Data are presented as n (%) or median (range), as available from the retrospective dataset. Percentages were calculated using the total study population as the denominator (N = 30), unless otherwise specified. Post-PGE1 SpO₂ was summarized only among the 22 patients who received PGE1 infusion. * Patients may have more than one comorbidity. DSA: digital subtraction angiography, PA-VSD: pulmonary atresia with ventricular septal defect, PGE1: prostaglandin E1, SpO₂: peripheral oxygen saturation

Characteristic	n (%) or median (range)
Age at admission, days	92 (1-173)
Weight, kg	3.6 (2-9.5)
Sex	
Male	10 (33.3%)
Female	20 (66.7%)
Gestational age	
Term	30 (100.0%)
Preterm	0 (0.0%)
Prenatal diagnosis	
Yes	0 (0.0%)
No	30 (100.0%)
Reason for admission	
Cyanosis	30 (100.0%)
Comorbidities*	
Pneumonia	12 (40.0%)
Sepsis	2 (6.7%)
Transposition of the great arteries	1 (3.3%)
None	16 (53.3%)
SpO₂ before PGE1, %	66 (40-90)
SpO₂ before <75%	23 (76.7%)
SpO₂ before ≥75%	7 (23.3%)
PGE1 infusion administered	22 (73.3%)
SpO₂ after PGE1, %, among PGE1-treated patients	85 (60-93)

The median age at admission was 92 days (range: 1-173 days), and the median body weight was 3.6 kg (range: 2-9.5 kg). There were 20 female patients (66.7%) and 10 male patients (33.3%). All patients were born at term, none had a prenatal diagnosis, and all were admitted because of cyanosis.

Comorbidities were documented in 14 patients (46.7%), most commonly pneumonia in 12 patients (40.0%), followed by sepsis in two patients (6.7%) and transposition of the great arteries in one patient (3.3%). Sixteen patients (53.3%) had no recorded comorbidities. The patient with transposition of the great arteries was retained in the analysis because the anomaly did not prevent identification of DA origin, course, number, or diameters on echocardiography or DSA and was not considered to substantially alter DA morphology for this study.

Before PGE1 administration, the median SpO₂ was 66% (range: 40-90%), with SpO₂ <75% in 23 patients (76.7%) and ≥75% in seven patients (23.3%). PGE1 infusion was administered in 22 patients (73.3%). Among these 22 treated patients, the median post-PGE1 SpO₂ was 85% (range: 60-93%).

Additional anatomical characteristics relevant to PA-VSD severity, including pulmonary artery confluence, MAPCAs, right-sided aortic arch, and branch pulmonary artery hypoplasia, were not consistently available in the retrospective dataset and therefore could not be summarized reliably in the Results.

Morphological characteristics of the DA on echocardiography

Echocardiographic findings of DA morphology are summarized in Table 2.

**Table 2:** Morphological characteristics of the DA on echocardiography Data are presented as n (%) or mean ± standard deviation, as appropriate. Percentages were calculated using the total study population as the denominator (N = 30). DA: ductus arteriosus

Characteristic	Echocardiography, N = 30
DA origin	
Brachiocephalic artery	11 (36.7%)
Subclavian artery	3 (10.0%)
Descending aorta	16 (53.3%)
DA orientation	
Vertical	17 (56.7%)
Horizontal	13 (43.3%)
DA diameter, mm	
Aortic end	3.12 ± 0.97
Pulmonary end	2.18 ± 0.62
Number of DAs	
Single	30 (100.0%)
Multiple	0 (0.0%)

On echocardiography, the DA most commonly originated from the descending aorta in 16 patients (53.3%), followed by the brachiocephalic artery in 11 patients (36.7%) and the subclavian artery in three patients (10.0%). Vertical DA orientation was observed in 17 patients (56.7%), while horizontal orientation was observed in 13 patients (43.3%). The mean DA diameter was 3.12 ± 0.97 mm at the aortic end and 2.18 ± 0.62 mm at the pulmonary end. All 30 patients (100.0%) had a single DA on echocardiography.

Morphological characteristics of the DA on DSA

Angiographic findings based on DSA are presented in Table 3.

**Table 3:** Morphological characteristics of the DA on DSA Data are presented as n (%) or mean ± standard deviation, as appropriate. Percentages were calculated using the total study population as the denominator (N = 30). DA: ductus arteriosus, DSA: digital subtraction angiography

Characteristic	DSA, N = 30
DA origin	
Brachiocephalic artery	12 (40.0%)
Subclavian artery	3 (10.0%)
Descending aorta	15 (50.0%)
DA orientation	
Vertical	17 (56.7%)
Horizontal	13 (43.3%)
DA diameter, mm	
Aortic end	3.86 ± 1.43
Pulmonary end	1.98 ± 0.83
Number of DAs	
Single	30 (100.0%)
Multiple	0 (0.0%)

On DSA, the DA most commonly originated from the descending aorta in 15 patients (50.0%), followed by the brachiocephalic artery in 12 patients (40.0%) and the subclavian artery in three patients (10.0%). Vertical DA orientation was observed in 17 patients (56.7%), while horizontal orientation was observed in 13 patients (43.3%). The mean DA diameter was 3.86 ± 1.43 mm at the aortic end and 1.98 ± 0.83 mm at the pulmonary end. All 30 patients (100.0%) had a single DA on DSA.

Comparison between echocardiography and DSA

Comparative results between echocardiography and DSA are shown in Table 4.

**Table 4:** Comparison of DA morphology between echocardiography and DSA Data are presented as n (%) or mean ± standard deviation, as appropriate. Percentages were calculated using the total study population as the denominator (N = 30). Paired continuous variables were compared using paired-samples t-tests. DA origin was compared using the McNemar-Bowker test for paired categorical variables with more than two categories. Non-significant p-values were not interpreted as evidence of diagnostic agreement or interchangeability. * DA orientation was not formally tested because independent assessment on both modalities showed exact concordance in 30/30 patients (100.0%). ** DA number was not formally tested because all patients had a single DA on both modalities, corresponding to exact concordance in 30/30 patients (100.0%). DA: ductus arteriosus, DSA: digital subtraction angiography

Characteristic	Echocardiography, N = 30	DSA, N = 30	Statistical test	Test statistic	p-value
DA diameter, mm					
Aortic end	3.12 ± 0.97	3.86 ± 1.43	Paired-samples t-test	t = 3.18	0.001
Pulmonary end	2.18 ± 0.62	1.98 ± 0.83	Paired-samples t-test	t = 1.32	0.198
DA origin			McNemar-Bowker test	χ² = 1.00	0.608
Brachiocephalic artery	11 (36.7%)	12 (40.0%)			
Subclavian artery	3 (10.0%)	3 (10.0%)			
Descending aorta	16 (53.3%)	15 (50.0%)			
DA orientation			Not tested*	Not applicable	Not applicable
Vertical	17 (56.7%)	17 (56.7%)			
Horizontal	13 (43.3%)	13 (43.3%)			
Number of DAs			Not tested**	Not applicable	Not applicable
Single	30 (100.0%)	30 (100.0%)			
Multiple	0 (0.0%)	0 (0.0%)			

The distribution of DA origin did not differ significantly between echocardiography and DSA (McNemar-Bowker test, χ² = 1.00, p = 0.608). However, this non-significant result was not interpreted as evidence of diagnostic agreement or interchangeability. Based on the available aggregate data, the marginal distributions differed by one case between echocardiography and DSA, with one fewer descending aortic origin and one additional brachiocephalic artery origin on DSA. Individual-paired discordance in DA origin could not be characterized from the aggregate dataset.

DA orientation was assessed independently on echocardiography and DSA using the same predefined ≥45° cutoff. Exact concordance for DA orientation was observed in 30/30 patients (100.0%), with vertical orientation in 17 patients (56.7%) and horizontal orientation in 13 patients (43.3%) on both modalities. All patients had a single DA on both modalities, corresponding to exact concordance of 30/30 patients (100.0%) for DA number.

The mean aortic-end DA diameter was significantly larger on DSA than on echocardiography (3.86 ± 1.43 mm vs. 3.12 ± 0.97 mm; paired-samples t-test, t = 3.18, p = 0.001). The mean pulmonary-end DA diameter did not differ significantly between DSA and echocardiography (1.98 ± 0.83 mm vs. 2.18 ± 0.62 mm; paired-samples t-test, t = 1.32, p = 0.198). DA diameter measurements were reported as absolute values in millimeters and were not indexed to body weight or body surface area.

Because individual paired diameter measurements were not available in the aggregate dataset, scatterplots, Bland-Altman plots, and intraclass correlation coefficients could not be generated. Therefore, the comparison of DA diameters should be interpreted as an exploratory paired comparison of mean differences rather than a formal measurement agreement analysis.

## Discussion

This study described DA morphology in patients with PA-VSD and, in an exploratory manner, assessed paired differences between echocardiography and DSA. The main findings were that DA morphology was variable; the descending aortic origin was the most common pattern; vertical orientation was observed in 17 patients (56.7%); and all 30 patients (100.0%) had a single DA. DSA demonstrated statistically larger aortic-end measurements than echocardiography, whereas pulmonary-end measurements did not differ significantly between modalities. Importantly, this study was not designed to establish diagnostic agreement, equivalence, or interchangeability between echocardiography and DSA.

The median age at admission was 92 days, indicating that most patients were evaluated after the early neonatal period. This finding has important clinical implications because PA-VSD is a duct-dependent or collateral-dependent cyanotic congenital heart disease in which delayed recognition may expose patients to worsening cyanosis, ductal constriction, infection-related deterioration, and delayed referral for catheter-based or surgical palliation [2,8]. None of the 30 patients (100.0%) had a prenatal diagnosis. This finding may reflect limitations in fetal cardiac screening, referral pathways, or access to specialized prenatal echocardiography in the study setting. Although this study did not evaluate outcomes related to delayed diagnosis, the absence of prenatal detection highlights the need to strengthen antenatal screening and early postnatal referral for complex congenital heart disease.

Female patients accounted for 66.7% of the cohort. This predominance should be interpreted cautiously because the sample size was small and the study was conducted at a single tertiary referral center. The observed sex distribution may reflect chance variation, referral patterns, or selection bias rather than a true sex-specific difference in PA-VSD occurrence. Larger multicenter cohorts would be required to determine whether this distribution differs meaningfully from broader congenital heart disease populations.

The most common DA origin in this cohort was the descending aorta, observed in 16 patients (53.3%) on echocardiography and 15 patients (50.0%) on DSA, followed by the brachiocephalic artery and subclavian artery. This finding is broadly consistent with previous anatomical descriptions showing that DA morphology in pulmonary atresia and other complex congenital heart diseases can be highly variable [1,5]. However, direct comparison with previous PA-VSD cohorts is limited because many published studies focus on angiographic classification, ductal stenting outcomes, or mixed duct-dependent pulmonary circulation rather than quantitative DA origin and orientation in PA-VSD alone [3-5,7,9]. Therefore, the predominance of descending aortic origin in this study should be interpreted as a descriptive finding from a local cohort rather than as a definitive anatomical distribution for PA-VSD.

Vertical DA orientation was observed in 17 patients (56.7%) on both echocardiography and DSA. Previous studies have emphasized that DA course, angulation, and tortuosity influence the technical feasibility and outcomes of ductal stenting [3,4,9,10]. A vertical or sharply angulated DA may make catheter alignment, guidewire passage, stent tracking, and complete ductal coverage more difficult. It may also increase the risk of procedural instability, incomplete coverage of the ductal tissue, residual stenosis, stent migration, or vascular injury. The frequency of vertical orientation in this cohort suggests that a substantial proportion of patients may have ductal anatomy requiring careful procedural planning. However, the locally defined ≥45° threshold for classifying vertical versus horizontal DA orientation has not been externally validated. Therefore, orientation results should be interpreted descriptively and should not be treated as a validated risk classification.

The distribution of DA origin did not differ significantly between echocardiography and DSA (McNemar-Bowker test, χ² = 1.00, p = 0.608). However, the absence of a statistically significant difference does not establish diagnostic agreement or interchangeability. Hypothesis testing evaluates whether marginal distributions differ, whereas agreement analysis evaluates whether the same patient receives the same classification across modalities. Because formal agreement statistics such as Cohen’s kappa were not performed, the categorical comparison should be interpreted only as an exploratory comparison of modality-specific findings. Similarly, exact concordance between DA orientation and DA number supports descriptive consistency in this cohort but, by itself, does not establish broader diagnostic reproducibility.

The small difference in DA origin classification between echocardiography and DSA may reflect several technical and anatomical factors. Echocardiography may be limited by acoustic windows, ductal angulation, small patient size, overlap with adjacent arch branches, and difficulty visualizing the full extracardiac ductal course. DSA may better delineate the ductal origin and systemic arterial connections through selective angiographic projection and contrast opacification. Conversely, DSA interpretation can also be influenced by projection angle, vessel overlap, catheter position, and contrast filling dynamics. Therefore, discordant DA origin classification, when present, should not be interpreted simply as an error by one modality, but rather as a reflection of the different technical strengths and limitations of each imaging method.

DSA demonstrated statistically larger aortic-end DA measurements than echocardiography. Several mechanisms may explain this difference. DSA provides direct angiographic opacification of the ductal lumen and may better define the aortic ampulla, especially when the DA has an oblique, vertical, or tortuous course. Contrast injection may transiently distend the ductal lumen, while magnification calibration, projection angle, and selection of the frame with maximal opacification may influence the measured diameter. Additionally, hemodynamic conditions during catheterization, sedation or anesthesia, PGE1 exposure, and the interval between echocardiography and DSA may affect vessel caliber. Echocardiography may underestimate the aortic end-diastolic diameter due to oblique imaging planes, suboptimal acoustic windows, and incomplete visualization of the aortic insertion.

Although the aortic-end difference was statistically significant, its clinical significance remains uncertain. The observed difference may be relevant to procedural planning because ductal diameter can influence stent diameter selection, landing-zone assessment, and the risk of incomplete coverage or residual narrowing. However, this study did not directly evaluate stent sizing decisions, procedural success, complications, reintervention, or post-stenting pulmonary artery growth. Therefore, the larger aortic-end measurements on DSA should be interpreted as a modality-related measurement difference with potential procedural relevance, not as definitive evidence that DSA improves clinical outcomes.

The role of DSA should be interpreted in the context of evolving imaging strategies. DSA remains clinically important because it provides real-time, high-resolution vascular mapping during catheterization and can directly guide interventional planning. However, this study should not be read as establishing DSA as an absolute reference standard. Advanced non-invasive imaging, particularly CT angiography and cardiac MRI, can provide additional information on aortic arch anatomy, MAPCAs, pulmonary artery confluence, and branch pulmonary artery hypoplasia. The absence of routine CT angiography or cardiac MRI in this study limited the comprehensive assessment of extracardiac vascular anatomy and may have affected the interpretation of DSA-based findings. In this context, DSA is better considered a complementary modality for selected patients rather than a definitive reference standard.

All patients in this study had a single DA. Although rare cases of multiple DAs have been reported [11], the absence of multiple DAs in this cohort may reflect the small sample size, local referral patterns, or the anatomical profile of patients who underwent both echocardiography and DSA. Because rare morphologic variants are clinically important for procedural planning, larger multicenter studies are needed to estimate their true frequency and clinical significance.

Limitations

This study has several limitations. First, the sample size was small because PA-VSD is a rare congenital heart disease, limiting statistical power and making the study underpowered for subgroup analyses and rare morphologic variants. Second, this was a retrospective single-center study, and only patients who underwent both echocardiography and DSA were included. This may have introduced referral and selection bias because patients undergoing DSA may have been more likely to require invasive anatomical assessment or to be considered for catheter-based or surgical intervention.

Third, the total number of PA-VSD patients screened during the study period and detailed exclusion counts could not be reliably reconstructed from the retrospective dataset; therefore, a complete screening flow diagram could not be provided. Fourth, additional anatomical features relevant to PA-VSD severity, including pulmonary artery confluence, right-sided aortic arch, and branch pulmonary artery hypoplasia, were not consistently documented and could not be fully analyzed. No patient with confirmed MAPCAs was included in the final analysis.

Fifth, image review was retrospective, and complete blinding to findings from the alternate modality could not be guaranteed because both imaging studies were used clinically during patient care. Additionally, echocardiographic images were reviewed by one pediatric cardiologist and DSA images by one pediatric interventional cardiologist; therefore, interobserver agreement and intraobserver reproducibility could not be assessed. Sixth, formal diagnostic agreement analyses, including Cohen’s kappa, Bland-Altman analysis, and intraclass correlation coefficients, were not performed. As a result, the findings cannot establish diagnostic agreement, measurement agreement, equivalence, or interchangeability between echocardiography and DSA.

Seventh, the locally defined ≥45° cutoff for vertical DA orientation was a pragmatic operational definition and has not been externally validated. Eighth, the timing of echocardiography and DSA relative to PGE1 administration, sedation or anesthesia during DSA, hemodynamic conditions, and the interval between imaging studies may have influenced ductal caliber and morphology. Ninth, DA diameters were reported as absolute measurements and were not indexed to body weight or body surface area, despite variation in age and weight. Finally, routine CT angiography or cardiac MRI was not available for comparison, which limited the comprehensive assessment of extracardiac vascular anatomy and prevented DSA from being interpreted as an absolute reference standard in this study.

## Conclusions

DA morphology in patients with PA-VSD was variable, with descending aortic origin and vertical orientation being common in this cohort. Echocardiography and DSA showed similar categorical distributions for DA origin, orientation, and number; however, these findings should not be interpreted as evidence of diagnostic agreement, equivalence, or interchangeability because formal agreement analyses were not performed.

DSA demonstrated significantly larger aortic-end DA measurements than echocardiography, while pulmonary-end measurements did not differ significantly between modalities. The clinical impact of this aortic-end measurement difference on stent sizing, procedural success, complications, reintervention, or patient outcomes was not directly evaluated.

Future prospective studies should use standardized echocardiographic, DSA, and preferably CT angiographic protocols; blinded independent image review; interobserver and intraobserver reproducibility assessment; and formal agreement analyses such as Cohen’s kappa, Bland-Altman analysis, and intraclass correlation coefficients. These studies should also link imaging findings to procedural outcomes, including feasibility of ductal stenting, stent size selection, technical success, complications, reintervention, and pulmonary artery growth.
